# *In vitro* evidence for senescent multinucleated melanocytes as a source for tumor-initiating cells

**DOI:** 10.1038/cddis.2015.71

**Published:** 2015-04-02

**Authors:** C Leikam, A L Hufnagel, C Otto, D J Murphy, B Mühling, S Kneitz, I Nanda, M Schmid, T U Wagner, S Haferkamp, E-B Bröcker, M Schartl, S Meierjohann

**Affiliations:** 1 Department of Physiological Chemistry I, University of Wurzburg, Biocenter, Am Hubland, Wurzburg, Germany; 2 Experimental Surgery, Experimental Transplantation Immunology, Clinic of General, Visceral, Vascular and Pediatric Surgery (Surgical Clinic I), University of Wurzburg Hospital, Wurzburg, Germany; 3 Institute of Cancer Sciences, Beatson Institute for Cancer Research, University of Glasgow, Glasglow, UK; 4 Institute for Human Genetics, University of Wurzburg, Biocenter, Am Hubland, Wurzburg, Germany; 5 Department of Dermatology, University Hospital Erlangen, Erlangen, Germany; 6 Department of Dermatology, Venereology and Allergology, University Hospital Wurzburg, Wurzburg, Germany; 7 Comprehensive Cancer Center Mainfranken, University Hospital Wurzburg, Wurzburg, Germany

## Abstract

Oncogenic signaling in melanocytes results in oncogene-induced senescence (OIS), a stable cell-cycle arrest frequently characterized by a bi- or multinuclear phenotype that is considered as a barrier to cancer progression. However, the long-sustained conviction that senescence is a truly irreversible process has recently been challenged. Still, it is not known whether cells driven into OIS can progress to cancer and thereby pose a potential threat. Here, we show that prolonged expression of the melanoma oncogene N-RAS^61K^ in pigment cells overcomes OIS by triggering the emergence of tumor-initiating mononucleated stem-like cells from senescent cells. This progeny is dedifferentiated, highly proliferative, anoikis-resistant and induces fast growing, metastatic tumors. Our data describe that differentiated cells, which are driven into senescence by an oncogene, use this senescence state as trigger for tumor transformation, giving rise to highly aggressive tumor-initiating cells. These observations provide the first experimental *in vitro* evidence for the evasion of OIS on the cellular level and ensuing transformation.

Cellular senescence is characterized by cell-cycle arrest and alterations in cell shape and metabolism, and can be triggered either by the sequential loss of telomeres or by numerous forms of cellular stress, for example, UV irradiation, oxidative stress or aberrant oncogenic signaling (premature senescence). In particular, oncogene-induced senescence (OIS), driven for example by activated RAS or BRAF, is an anti-cancer protection mechanism that prevents tumor generation despite the presence of oncogenic mutations. For instance, human nevi exhibit enhanced MAPK signaling caused by activating mutations in B-RAF or N-RAS. They display classical characteristics of senescence,^1^ and remain benign in the large majority of cases. However, nevi are also supposed to give rise to a quarter of all melanomas.^2^

Along the same lines, oncogenic RAS clearly triggers OIS in different cell types *in vivo*,^3, 4, 5, 6^ but activated RAS is detected in up to 30% of human cancers.^7, 8^ This indicates that senescence bypass is a key feature of cancer development. The fact that many premalignant tissues with tumorigenic potential display features of senescence has led to the concept that OIS precedes transformation, and tumors arise from senescent tissue.^1, 5, 6, 9^ However, as OIS was long considered to be irreversible, it was not clear how this transformation process can take place. Recently, there has been accumulating evidence that OIS can be reversed under certain circumstances on the cellular level. Specifically, H-RAS^12V^ induces senescence in fibroblasts, which is caused by ribonucleotide reductase (RRM2) suppression and accompanying dNTP reduction.^10^ Interestingly, the forced re-expression of RRM2 is able to overcome senescence. Still, is not known whether a senescent primary cell might give rise to cancer. While the later steps of tumor progression are fairly well understood, early events in tumorigenesis such as the transition of a benign senescent lesion to a tumor are still enigmatic.

Here, we reveal that long-term NRAS^61K^ activation in melanocytes triggers a strong senescent phenotype characterized by multinucleation, which then is followed by the post-senescence generation of tumor-initiating cells with stem cell-like properties. The results demonstrate that senescence in melanocytes can be overcome on the cellular level and can also be a source for malignant cancer cells.

## Results

### Multinucleated cells are frequently found in human nevi

**Figure 1:**
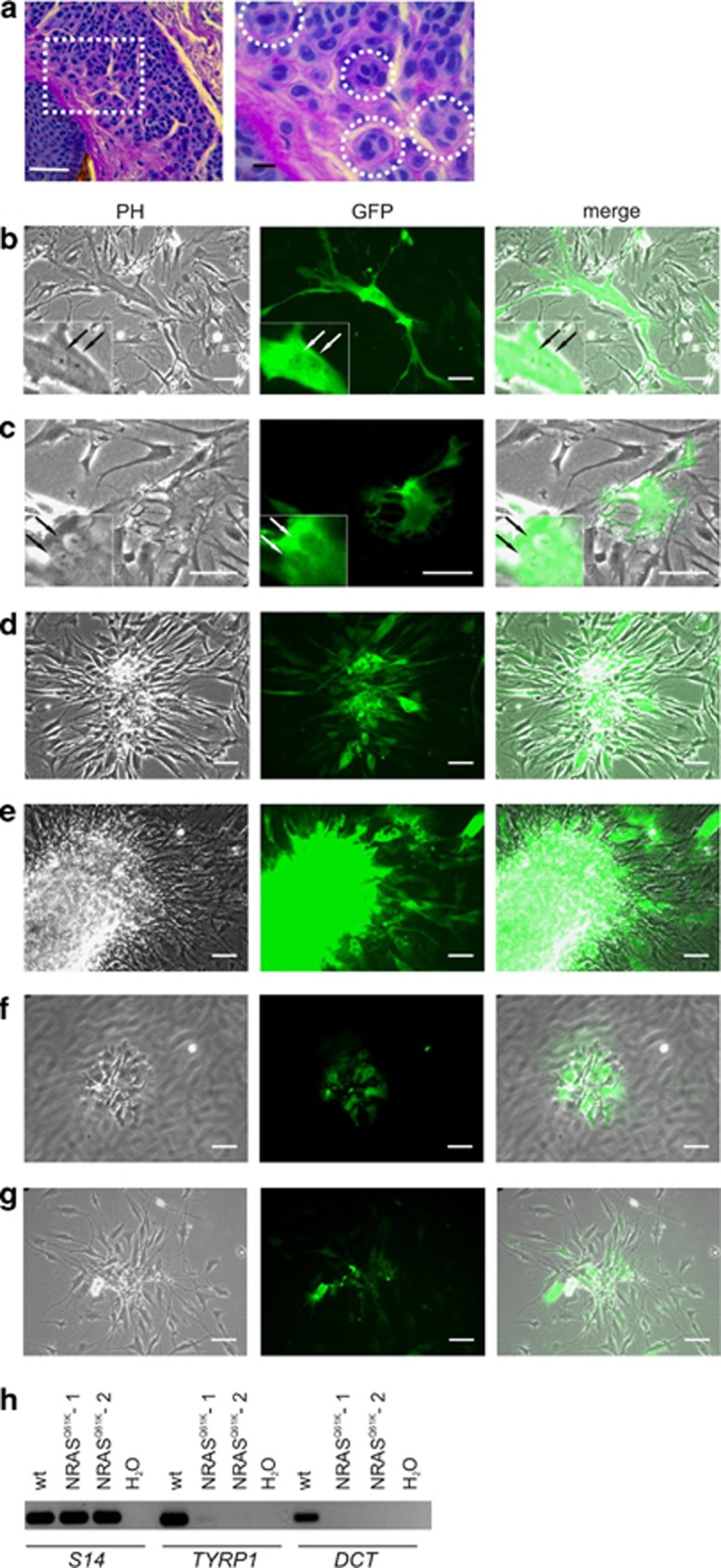
Nevus melanocytes and senescent melanocytes are frequently multinucleated. (**a**) Left: Acquired cutaneous nevus, stained with periodic acid Schiff (PAS) reagent to visualize the membranes. PAS was used for the detection of mucosubstances including glykoproteins and glykolipids that are characteristic for cellular membranes. The dense, dark stained lines between the cells demarcate the membranes. Right**:** Magnification of the highlighted region. Some of the multinucleated cells are marked by white circles. White scale bar: 25 *μ*m, black scale bar: 5 *μ*m. (**b** and **c**) Expression of N-RAS^61K^ using the plasmid pCDH-CMV-N-RAS^61K^-EF1-copGFP, inducing flat and binuclear appearance shortly after introduction of the plasmid. The arrows in (**b** and **c**) indicate two different nuclei in single cells. (**d** and **e**) After 3 weeks of cultivation, GFP/N-RAS^61K^-positive cells started to form colonies, which overgrew the cell culture. (**f**) In addition, detached cell clusters were found in the supernatant. The unfocused cells in the background represent the attached, non-transfected cells at the bottom of the culture dish. (**g**) The detached cell clusters could be replated, demonstrating anoikis resistance. Scale bars, 100 *μ*m. Similar observations were made in three independent experiments. (**h**) RT-PCR analysis (28 cycles) of *TYRP1* and *DCT* expression in NHEM control cells and two N-RAS^61K^-transgenic NHEM cell clones, which grew out after several weeks of cultivation and developed anoikis resistance. The RT-PCR is representative of three independent biological replicates. Ribosomal *S14* was used as a reference

When we analyzed randomly chosen sections of paraffin-embedded acquired melanocytic nevi, we found multinucleated nevus cells in 13/28 samples (46%). In most cases, samples contained few multinucleated cells, amounting to less than 1% of the nevus cell population, although some nevi showed a higher abundance of these abnormal cells, reaching up to 23% (Figure 1a). *In vitro*, such bi- or multinucleation of non-transformed cells occurs under conditions of senescence.^11, 12, 13, 14^ The strong nuclear disturbance induces an efficient cell-cycle block in non-transformed cells. The causes leading to multinucleation can be manifold and encompass UV damage, elevated levels of reactive oxygen species (ROS) and oncogenic stress, for example, by the melanoma oncogene N-RAS^61K^ that efficiently induces OIS in melanocytes.^11, 12, 14, 15^

To investigate the dual role of oncogenic RAS as senescence inducer and tumor driver, we overexpressed N-RAS^61K^ in a vector with simultaneous GFP coexpression in normal human epidermal melanocytes (NHEM). As expected, we observed an N-RAS^61K^ –induced stop in cell-cycle proliferation (Supplementary Figure S1) as well as the induction of bi- and multinucleation by N-RAS^61K^ (Figures 1b and c). Although OIS is widely considered to be a tumor-suppressive process, we found that cultivation of oncogene-expressing primary human melanocytes for more than 3 weeks led to the generation of proliferating cell clones (Figures 1d and e). The cells overgrew the non-transfected cells, and clusters of viable cells were found in the supernatant, indicating anoikis resistance (Figures 1f and g). These cells lacked melanocyte differentiation markers such as *DCT* and *TYRP1* (Figure 1h).

### Multinucleated melanocytes give rise to proliferation-competent progeny

**Figure 2:**
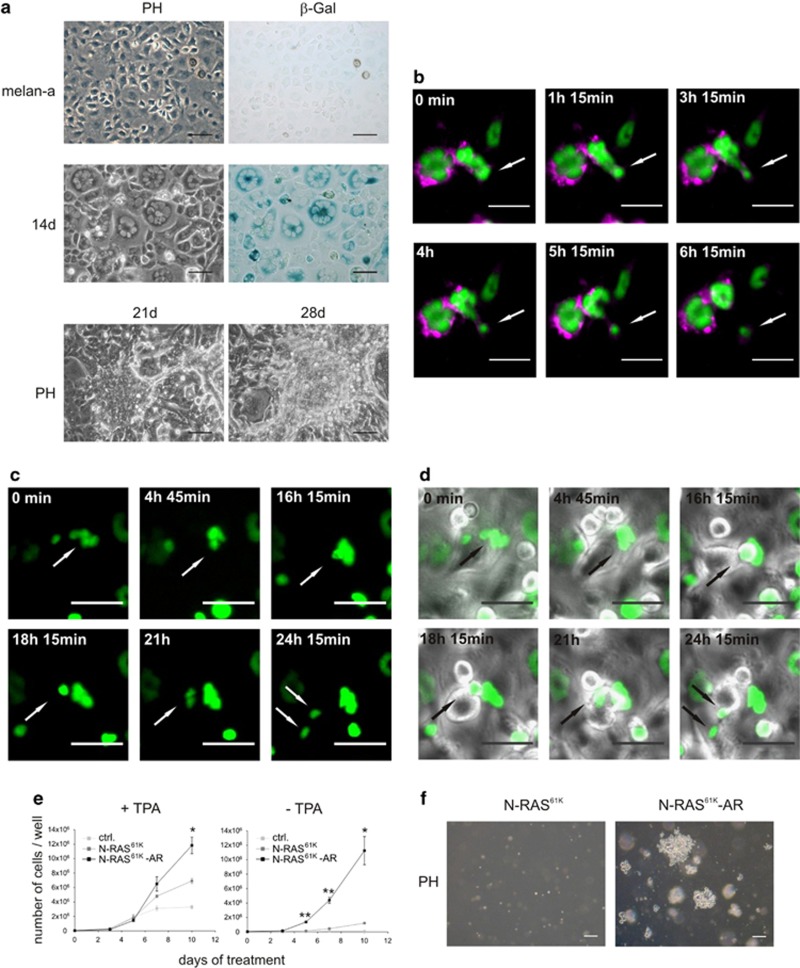
Multinuclear cells give rise to small proliferative cells. (**a**) Phase-contrast (PH) images of melan-a control cells and N-RAS^61K^ cells after 14, 21 and 28 days of doxycycline treatment (1 *μ*g/ml) and the corresponding brightfield images of SA-*β*-galactosidase-stained melan-a and N-RAS^61K^ cells after 14 days. Scale bars, 100 *μ*m. Please note that melan-a cells do not display signs of senescence in the absence of N-RAS^61K^. Similar observations were made in >5 independent experiments. (**b**) N-RAS^61K^ cells were transiently transfected with pBabe-MN (EF1a-red membrane and green nucleus)-2APuro before being plated onto glass coverslips. Upon 16 days of doxycycline treatment, cells were monitored for 17 h. Time points are indicated. Scale bars, 50 *μ*m. (**c** and **d**) N -RAS^61K^ cells were transiently transfected with pBabe-puro-H2-eGFP before being plated onto glass coverslips. Upon 27 days of doxycycline treatment, cells were monitored for 28 h at 100-fold magnification and pictures were taken every 15 min. Scale bars, 50 *μ*m. (**c**) GFP fluorescence of the cells. (**d**) As in (**c**), but merge of phase contrast and GFP. Time points are indicated. Arrows pinpoint the budding and dividing cell. (**e**) Proliferation of melan-a, N-RAS^61K^ and N-RAS^61K^-AR cells in DMEM containing 10% FCS, antibiotics and, where indicated, tetradecanoyl-12,13-phorbolacetate (TPA). (**f**) Soft agar growth assay of N-RAS^61K^ and N-RAS^61K^-AR cells, cultivated for 14 days in DMEM containing 10% FCS and antibiotics. Scale bars, 100 *μ*m. *P<0.05; **P<0.01

To follow the fate of multinucleated cells after longterm N-RAS^61K^ stimulation, we used murine instead of human melanocytes, as replicative exhaustion can be prevented in these cells under well-established *in vitro* culture conditions by the addition of tetradecanoyl-12,13-phorbolacetate (TPA).^16^ We have shown previously that N-RAS^61K^ expression in melan-a murine melanocytes similar to NHEM cells leads to a multinucleated phenotype. This is caused by N-RAS induction of ROS and is accompanied by p53 signaling and senescence-associated *β*-galactosidase (SA-*β*-Gal) expression.^11^ We used a doxycycline-inducible N-RAS^61K^ vector to drive long-term expression in melanocytes for extended time periods. After 14 days of doxycycline treatment, a large fraction of cells had become senescent, as demonstrated by their multinucleated phenotype and SA-*β*-Gal staining, which mostly correlated (Figure 2a, middle panel). In addition, proliferation was impeded, comparable to control cells which are not expected to proliferate in the absence of TPA (see Figure 5e: ‘ctrl' and ‘N-RAS^61K^'). Importantly, the overall levels of oncogenic N-RAS were only slightly enhanced (Supplementary Figure S2A), thus showing that this phenotype is visible under conditions which mimic the *in vivo* situation. N-RAS^61K^ expression went along with activation of the MAPK and PI3K pathways, as seen by enhanced P-ERK1/2 and P-AKT levels (Supplementary Figure S2B). The N-RAS^61K^ mediated senescence is characterized by activation of the p53 pathway, as indicated by p19-ARF induction as well as enhanced DNA damage signaling, which was visible as enhanced *γ*-H2AX and P-p53 levels. p21 levels were barely altered and p16 levels were not visible (Supplementary Figure S2B). After 2–3 weeks, we observed that multinucleated cells were sometimes surrounded by small mononucleated cells (Supplementary Figure S2C). Furthermore, after 3–4 weeks of N-RAS^61K^ expression, proliferating cells appeared that overgrew the cell culture and formed three-dimensional cellular aggregates typical for transformed cells *in vitro* (Figure 2a, lower panel). Concurrently, we noted the appearance of viable, detached cells in the culture supernatant. Replating of such floating cells was followed by reattachment before they again gave rise to detached cells. We termed these cells ‘N-RAS^61K^-AR' (for ‘anoikis resistant').

To trace the origin of the proliferating cells, we conducted time lapse movies of multinucleated N-RAS^61K^ cells after long-term oncogene stimulation. To resolve single cells, we transfected the N-RAS^61K^ cells with a plasmid, which allows to separately visualize membranes (red-purple) and nucleus (green). We recorded several highly multinucleated cells performing asynchronous cytokinesis, as for example shown in Figure 2b. The figure depicts two adjacent multinucleated cells with cytokinesis occurring in the cell on the right (Figure 2b, Supplementary Movie 1). Strikingly, this asynchronous cytokinesis resulted in the budding of small, mononuclear, viable cells that underwent further cell divisions (Figures 2c and d, Supplementary Movies S2A and B). The parental cells remained multinuclear and persisted throughout the experiments.

To compare the features of naïve N-RAS^61K^ cells, N-RAS^61K^-AR cells that arise after senescence induction, and melan-a control cells (transfected with pTRE2hyg control vector, but simply termed ‘melan-a' hereafter) we monitored proliferation under different cell culture conditions. N-RAS^61K^-AR cells but not melan-a or N-RAS^61K^ cells grew efficiently in the absence of TPA (Figure 2e) and under growth factor-deprived conditions (Supplementary Figures S3A and B).

The unexpected proliferation behavior of N-RAS^61K^-AR cells derived from senescent cell cultures was further confirmed by their capacity to efficiently form soft agar colonies (Figure 2f). Furthermore, when we analyzed four different N-RAS^61K^-AR cell clones, we noted several chromosomal aberrations, indicating that the cells had undergone a phase of genetic instability (Supplementary Table S1).

### N-RAS^61K^-AR cells are tumorigenic and dedifferentiated

**Figure 3:**
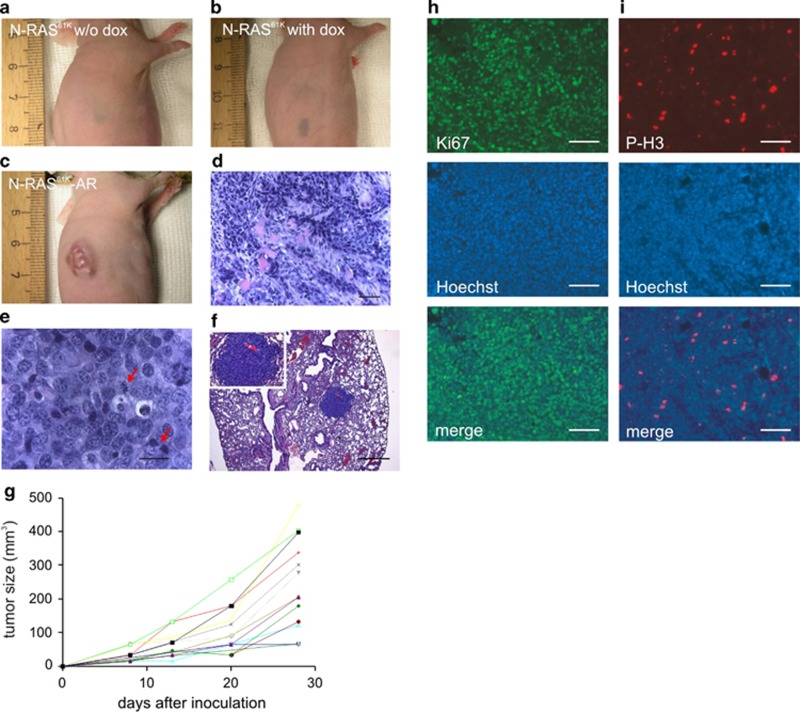
Long-term N-RAS^61K^ activation leads to melanocyte senescence followed by anoikis resistance and tumorigenicity *in vivo.* (**a** and **b**) Macroscopic appearance of subcutaneous tissue 10 weeks after injection of N-RAS^61K^ cells into nude mice. Where indicated, 2 mg/ml doxycycline (Dox) was added to the drinking water of the mice. (**c**) Macroscopic view of tumor development after subcutaneous injection of N-RAS^61K^ -AR cells into nude mice. (**d–f**) Hematoxylin/eosin stained tissue sections. In (**d** and **e**), N-RAS^61K^ -AR-derived tumors are displayed (**d**: scale bar, 50 *μ*m; **e**: scale bar, 20 *μ*m). The arrows in (**e**) indicate mitotic figures. (**f**) Lung metastasis in a N-RAS^61K^-AR-injected nude mouse. The inlay shows a magnification of the metastasis. Scale bar, 500 *μ*m. (**g**) Time-dependent development of subcutaneous tumors in N-RAS^61K^-AR-injected nude mice. The different colors represent the single tumors from both flanks of each mice. (**h**) Hoechst and Ki67 staining and (**i**) Hoechst and P-H3 staining of a tumor section from N-RAS^61K^-AR-injected mice visualizing strong mitotic activity throughout the tumor. Scale bars, 100 *μ*m

To analyze tumorigenicity *in vivo*, melan-a, N-RAS^61K^ and N-RAS^61K^-AR cells were injected subcutaneously into nude mice. Melan-a and N-RAS^61K^ cells gave rise to subcutaneous, pigmented, nevus-like structures (Figures 3a and b, Supplementary Figures S4A and B, Supplementary Table S2). N-RAS^61K^-AR cells, however, formed large tumors at all injection sites after approximately 10 days (Figures 3c, d–g,Supplementary Table S2). Strikingly, tumor formation occurred in the absence of doxycycline. Mitotic figures were abundant in tumor sections (Figure 3e), and staining of the S-phase marker Ki67 as well as the mitosis marker phospho-histone H3 (Ser10) was readily detectable in tumors, but not in nevus-like structures of control mice (Figures 3h and i, Supplementary Figures S4C and D), strongly indicating a high *in-vivo* proliferation potential of N-RAS^61K^-AR cells. The mice had to be killed after 4 weeks due to the high tumor load. Notably, by this time, the primary tumor had already metastasized to the lung (Figure 3f).

**Figure 4:**
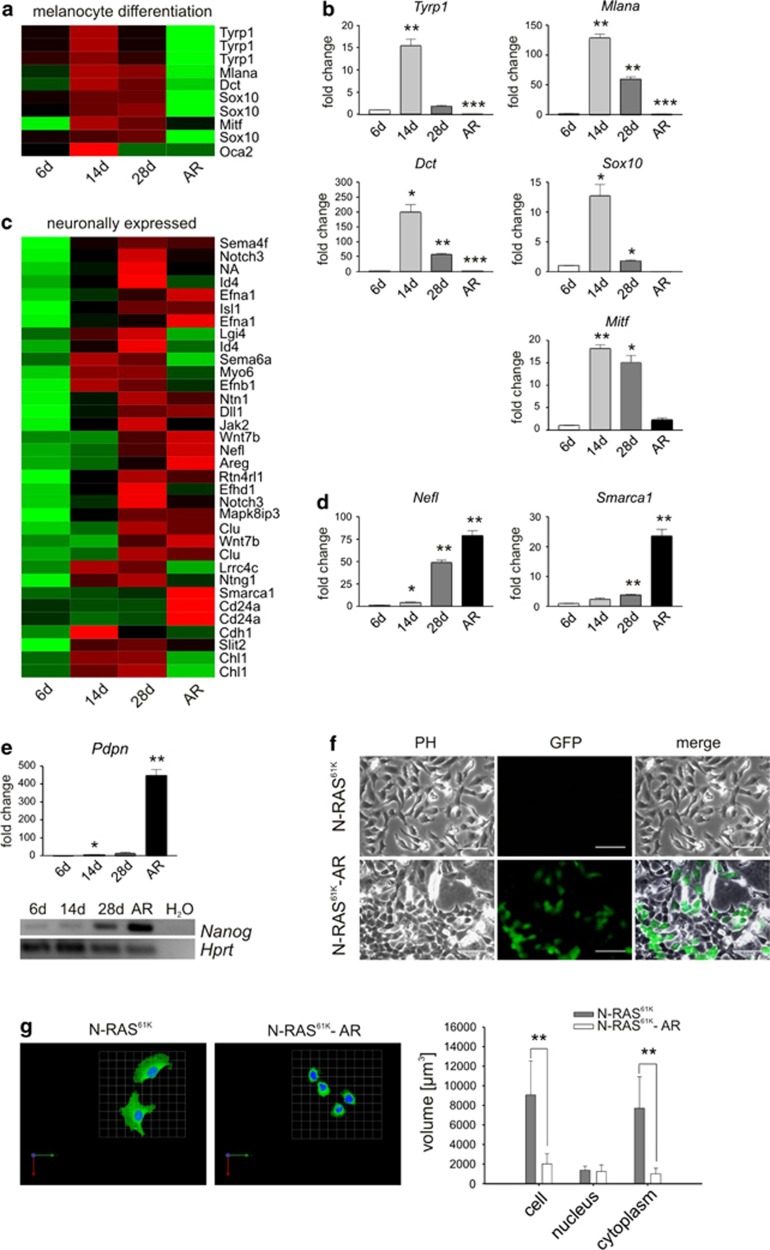
Long-term N-RAS^61K^ expression diminishes melanocyte marker expression and increases neuronal gene expression and pluripotency features. (**a**) Heatplot displaying the RNA expression of melanocyte differentiation markers in N-RAS^61K^ cells stimulated with doxycycline for 6, 14 and 28 days and in N-RAS^61K^-AR cells. (**b**) Confirmation of differential gene expression by real-time PCR using primers directed against *Tyrp1*, *Mlana*, *Dct*, *Sox10* and *Mitf.* (**c**) Heatplot displaying the RNA expression of neuronal genes in the same samples as described in (**a**). (**d**) Real-time analysis showing differential expression of *Nefl* and *Smarca.* For (**a** and **c**), the values are color coded using a green–red scale, where green indicates low expression and red indicates high expression. (**e**) Real-time PCR analysis of *Pdpn* expression (upper panel) and RT-PCR analysis of *Nanog* expression (40 cycles, lower panel) in N-RAS^61K^ cells stimulated with doxycycline for 6, 14 and 28 days and in N-RAS^61K^-AR cells. *Hprt* served as a control. (**f**) *Nanog*-driven GFP expression in N-RAS^61K^ and N-RAS^61K^-AR cells, transiently transfected with the PL-SIN-EOS-S(4+)-eGFP vector. Scale bars, 100 *μ*m. (**g**) Determination of the nuclear/cytoplasmic ratio of N-RAS^61K^ and N-RAS^61K^–AR cells. Left**:** Confocal images of N-RAS^61K^ and N-RAS^61K^-AR cells. Arrows, 50 *μ*m. Right: Averaged cellular, nuclear or cytoplamic volume of N-RAS^61K^ and N-RAS^61K^-AR cells upon generation of confocal stacks (see Supplementary Movie S3). Compartment volumes were determined using Volocity v4 software, *P*=0.0076 (cell), *P*=0.0052 (cytoplasm); *n*=6. *P<0.05; **P<0.01, ***P<0.001

To gain insight into the process leading to the generation of the highly aggressive N-RAS^61K^-AR cells from a previously senescent cell culture, expression profiling of N-RAS^61K^ cells at different times of doxycycline stimulation and of N-RAS^61K^-AR cells was performed. We found the expression of melanocyte differentiation markers to be highly regulated in response to N-RAS^61K^ expression (Figures 4a and b). Concurrent with senescence progression, differentiation markers, such as *Tyrp1*, *Mlana*, *Dct*, *Sox10* and *Mitf*, were strongly induced after 14 days. However, expression of most differentiation markers decreased after 28 days of N-RAS^61K^ induction and in N-RAS^61K^-AR cells. In contrast, *Mitf* levels were still higher in N-RAS^61K^-AR when compared with the 6-day sample. It is known that a certain level of MITF is similarly maintained in human melanoma, consistent with its role for melanocyte and melanoma proliferation and survival.^17^ Concomitant with the decrease of differentiation genes, numerous genes, which are typical for neuronal tissues or the neural crest, were induced (Figures 4c and d). Many of these genes were still expressed in N-RAS^61K^-AR cells. During embryonic development, melanocytes arise from the neural crest lineage. Re-expression of embryonic or neuronal genes is associated with a stem cell-like phenotype and is a hallmark of aggressive human melanomas.^18, 19^ To test whether the N-RAS^61K^-AR cells display features of stem-like cells, we looked at the expression of embryonal and stem cell markers. Interestingly, *Pdpn* and *Nanog* were among the genes whose expression was markedly increased in N-RAS^61K^-AR cells (Figure 4e). In particular, NANOG is considered as a master transcription factor for the maintenance of the undifferentiated state and cellular self-renewal. Using a NANOG-driven GFP reporter,^20^ we found a large fraction of N-RAS^61K^-AR cells with activated *Nanog* promoter activity, which was not observed in N-RAS^61K^ cells (Figure 4f). N-RAS^61K^-AR cells also displayed a reduction of cytoplasmic volume and consequently a higher nuclear/cytoplasmic ratio compared with N-RAS^61K^ cells (Figure 4g, Supplementary Movie S3), which is a morphological feature of stem-like and dedifferentiated cells.

Further analysis of the genes distinguishing N-RAS^61K^-AR cells from all other samples led to two prominent groups: (1) positive regulation of proliferation and (2) meiosis genes (Supplementary Figure S5). The group ‘positive regulation of proliferation' included numerous genes encoding growth factors (e.g., *Kitl*, *Vegfa*, *Hbegf*, *Btc* and *Areg*) and growth factor receptors (*Pdgfra*, *Flt1*) as well as enzymes involved in generation and transmission of growth-promoting signaling molecules (*Ptgs2, Fabp4*) (Supplementary Figure S5A and B). These data suggest that the AR cells are able to provide a number of autocrine signals, which sustain cell growth even under growth-restricting conditions such as starvation or withdrawal of TPA (as reported above). Indeed, ERK1/2 activity, which efficiently transmits growth-promoting signals in melanocytes, was increased in N-RAS^61K^-AR cells and reached higher levels than in doxycycline-stimulated N-RAS^61K^ cells (Supplementary Figure S5C).

Among the genes included in the meiosis signature (Supplementary Figures S5D and E), *Spo11* is also known as cancer/testis gene^21^ and it displays copy number gains in different cancer types such as colorectal and gastric cancer (www.oncomine.org). *Cyp26b1* is involved in retinoic acid metabolism and has a role in neural crest-derived tissue development.^22^ Meiosis-related genes are often upregulated in melanoma cells that are particularly prone to so-called ‘meiomitosis', describing the partial expression of meiosis machinery in mitotic cells.^23^ Taken together, the expression profile provides evidence for melanocyte dedifferentiation, which is a typical feature of invasive melanoma cells,^24, 25, 26^ upon long-term N-RAS^61K^ activation and reveals the expression of stem-like and meiosis-associated genes in N-RAS^61K^-AR cells.

### The development of anoikis resistance is dependent on prior senescence

**Figure 5:**
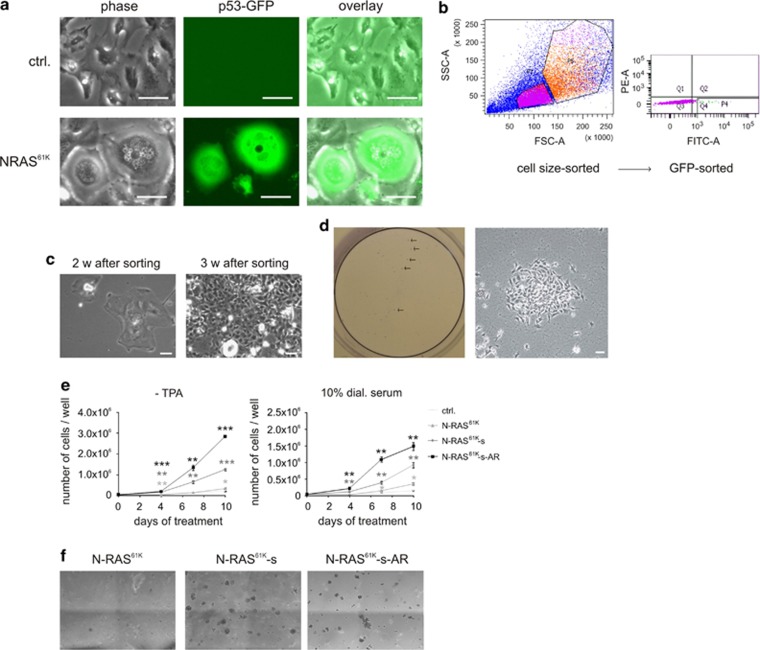
Only senescent NRAS^61K^ cells give rise to anoikis-resistant progeny. (**a**) Melan-a control cells (upper images) and N-RAS^61K^ cells (lower images) were cultivated in the presence of doxycycline (Dox) before they were transiently transfected with the pGreenFire-p53 reporter plasmid. Note the strong GFP staining for the multinucleated N-RAS^61K^ melanocytes. Scale bars, 75 *μ*m. (**b**) After 1 week of doxycycline stimulation, p53-GFP-transfected N-RAS^61K^ cells were sorted for large cell size (as characteristic for the senescent multinucleated cells) and strong GFP positivity. (**c**) Appearance of cells 2 weeks after sorting (left) and 1 week later (right). Scale bars, 35 *μ*m. (**d**) After 6 weeks, supernatant from sorted cells was transferred to a new dish and was allowed to attach overnight, before a crystal violet staining was performed. Please note that due to the low initial cell density in the dishes, the amount of cells in the supernatant is much lower than in previous figures, thus giving only rise to weak crystal violet staining. Arrows indicate the presence of some exemplary cell colonies (left). A magnification shows the accumulation of many cells to one colony, suggesting that the cells that gave rise to the colony were present as spheroid in the cell culture supernatant (right). Scale bar, 50 *μ*m. (**e**) Proliferation of melan-a (ctrl.), and unsorted N-RAS^61K^ cells (N-RAS^61K^) as well as N-RAS^61K^ cells sorted for GFP and large cell size (N-RAS^61K^-s) and corresponding anoikis-resistant cells from the supernatant (N-RAS^61K^-s-AR). Cells were either kept in starving medium (10% dialyzed FCS) containing Dox or in normal culture medium without TPA, as indicated. Asterisks indicate statistical significance of N-RAS^61K^-s and N-RAS^61K^-s-AR cells compared with N-RAS^61K^ cells. *P<0.05; **P<0.01, ***P<0.001; *n*=3. (**f**) Soft agar growth assay of N-RAS^61K^, N-RAS^61K^-s and N-RAS^61K^-s-AR cells, cultivated for 14 days in DMEM containing 10% FCS and antibiotics

To verify that the cell population that gives rise to anoikis-resistant cells is truly senescent, we made use of the fact that p53 is strongly induced during senescence in N-RAS^61K^-expressing multinucleated melanocytes.^11^ N-RAS^61K^ cells were transiently transfected with the p53-specific GFP reporter plasmid. The pGreenFire-p53 plasmid is a lentiviral reporter vector containing a p53 responsive element, thus directly displaying p53 activity. A strong GFP signal was visible in senescent multinucleated N-RAS^61K^ cells, but not in control melan-a cells (Figure 5a). To obtain a 100% senescent melanocyte population, N-RAS^61K^ cells were strictly sorted for high GFP intensity and large cell size by flow cytometry (Figure 5b) and were then seeded at low density. These isolated multinucleated cells were termed N-RAS^61K^-s (s for ‘sorted'). They were able to repopulate the culture dish with small cells after 3 weeks (Figure 5c). Furthermore, anoikis-resistant cells appeared after the cells became confluent (Figure 5d), confirming that they originate from the senescent cells. The anoikis-resistant cells were harvested from the supernatant (‘N-RAS^61K^-s-AR'), and their growth was monitored in comparison with N-RAS^61K^- and N-RAS^61K^-s cells. As expected, N-RAS^61K^-s and N-RAS^61K^-s-AR, but not the parental N-RAS^61K^ cells displayed a strong proliferation potential both in the absence of TPA and in growth factor-reduced serum (Figure 5e). In addition, they were able to efficiently grow in soft agar (Figure 5f).

**Figure 6:**
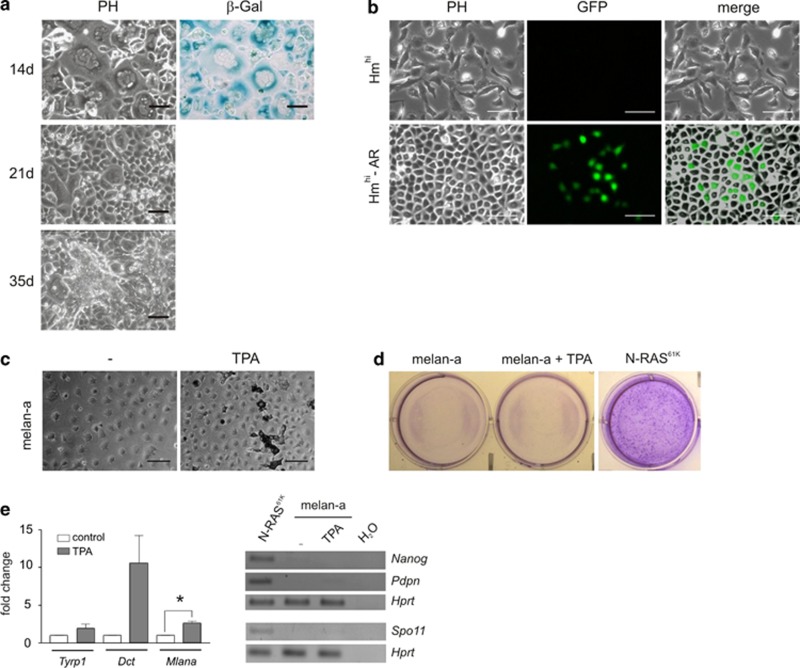
OIS is required for the pluripotent phenotype. (**a**) Phase-contrast (PH) images of unstained Hm^hi^ cells after 14, 21 and 35 days of EGF treatment (100 ng/ml) and brightfield images of SA-*β*-galactosidase-stained (*β*-Gal) Hm^hi^ cells after 14 days of EGF treatment. (**b**) *Nanog*-driven GFP expression (GFP) in Hm^hi^ and Hm^hi^-AR cells, transiently transfected with the PL-SIN-EOS-S(4+)-eGFP vector. Scale bars, 100 *μ*m. (**c**) Phase-contrast images of melan-a cells cultivated for 28 days without any additives (−) or in the presence of TPA. Scale bars, 100 *μ*m. (**d**) N-RAS^61K^ cells were cultivated for 28 days in the presence of doxycycline (Dox), whereas melan-a cells were kept in media with or without TPA. Upon 28 days of treatment, supernatant was transferred to a new 6-well plate, which was incubated for 24 h to allow cells to re-attach, followed by a 2% crystal violet staining. (**e**) Left panel**:** Real-time PCR analysis of *Tyrp1*, *Dct* and *Mlana* from melan-a cells cultivated for 28 days in the absence or presence of TPA, respectively. (**e**) Right panel**:** RT-PCR analysis of *Nanog, Spo11* (40 cycles each) and *Pdpn* (33 cycles) expression of melan-a cells starved for 28 days or treated with TPA, respectively. Dox-treated N-RAS^61K^ cells, also harvested after 28 days, served as a positive control. *Hprt* was used as a reference. *P<0.05

To test the effect of an independent OIS inducer, we used the melanoma-associated receptor tyrosine kinase Xmrk, an EGFR family protein that strongly drives MAPK and PI3K pathways and induces a senescence phenotype with accompanying proliferation inhibition similar to N-RAS^61K^.^11, 27^ Again, senescence of oncogene-expressing melan-a cells was followed by the generation of three-dimensionally growing cells and anoikis resistance (Figure 6a). Similar to the observation for N-RAS^61K^-AR cells, *Nanog* promoter activity was also induced by this oncogene (Figure 6b).

Senescence induced by oncogenes is not immediately executed after oncogene activation, but only comes into action after a previous proliferation boost caused by strong, growth-promoting signals.^28^ To determine whether a proliferation stimulus *per se* leads to the observed dedifferentiation and anoikis resistance, we cultivated the parental cell line melan-a for long time periods in the presence of the growth stimulus TPA, an efficient activator of PKC and the MAPK pathway. Apart from the addition of TPA, melan-a cells underwent the same treatment as N-RAS^61K^ cells. After TPA-treated melan-a cells had reached confluency, pigmentation increased (Figure 6c). Upon 28 days of treatment, we could neither observe viable, detached melan-a cells in the supernatant nor the three-dimensional cell colonies we observed in N-RAS^61K^ cells (Figures 6c and d). Furthermore, these cells showed the induction neither of the pluripotency markers *Pdpn* and *Nanog* nor of the male meiosis marker *Spo11,* whereas the melanocyte markers *Mlana*, *Tyrp1* and *Dct* were generally upregulated (though the data only reached significance in case of *Mlana*) (Figure 6e). Thus, the mere induction of proliferation is not sufficient to allow the generation of AR-cells.

### Anoikis resistance does not depend on oncogene expression levels

To investigate whether a change in N-RAS^61K^ expression could affect senescence phenotype or anoikis resistance, we generated an independent N-RAS^61K^ transgenic cell line, which allows very fine-tuned expression induction accompanied by inducible GFP fluorescence. We applied 10, 25, 100 and 1000 ng/ml doxycycline to these cells and followed their behavior over time. From 25 ng/ml on, expression was visible on GFP level (Supplementary Figure S6A) as well as *NRAS* RNA level (Supplementary Figure S6B). At 0 and 10 ng/ml, no GFP or RNA signals were detected. Interestingly, senescence was only visible in those cells, which were treated with doxycycline concentrations starting from 25 ng/ml (Supplementary Figure S7A). In accordance, all these cells developed anoikis resistance (Supplementary Figure S7B) and dedifferentiation as well as upregulation of the AR marker genes *Pdpn* and *Nefl* (Supplementary Figures S7C and E). Although a slight downregulation of differentiation genes was even detected after treatment with 10 ng/ml doxycycline, the stemness gene *Pdpn* and the neuronal gene *Nefl* were barely changed under these conditions. These data show that the degree of N-RAS^61K^ expression is not important for senescence and anoikis resistance once a critical, but small threshold level is reached.

### Signaling pathways involved in senescence-dependent anoikis resistance

**Figure 7:**
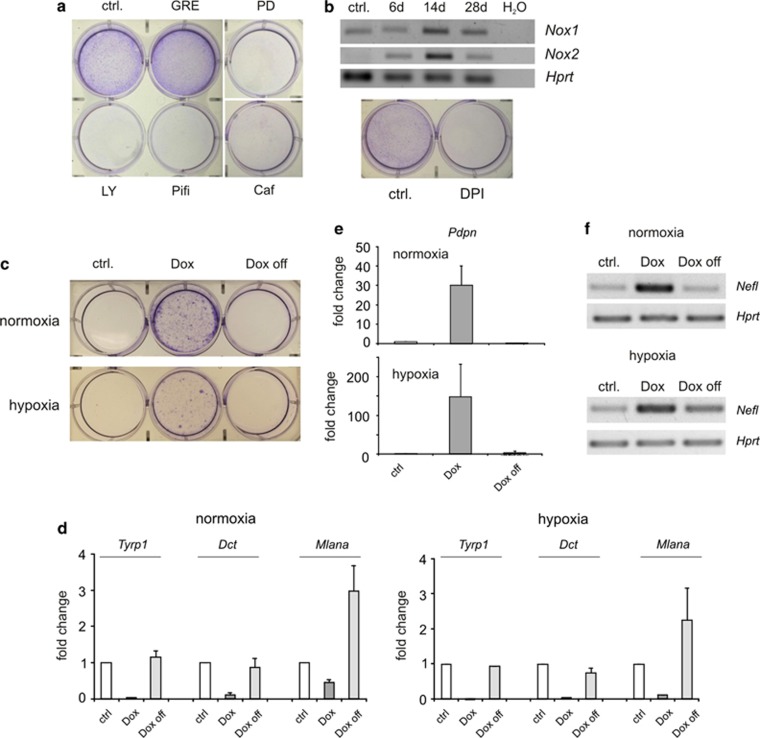
Involvement of signaling pathways in N-RAS^61K^-AR generation. (**a**) N-RAS^61K^ cells were cultivated for 4 weeks in the presence of doxycycline (Dox) and DMSO (ctrl.), the PI3K inhibitor LY294002 (LY, 10 *μ*M), the antioxidant glutathione reduced ethyl ester (GRE, 1 mM), the p53 inhibitor pifithrine (Pifi, 10 *μ*M), the MEK inhibitor PD184352 (PD, 2 *μ*M) or the ATM inhibitor caffeine (Caf, 1 mM). All agents were added at the beginning of the experiments and medium with inhibitors was replaced twice weekly. Scale bars, 100 *μ*m. (**b**) Upper image: RT-PCR analysis of NADPH oxidase isoforms *Nox1 and Nox2* in response to N-RAS^61K^ induction by doxycycline (Dox) (40 cycles). *Hprt* served as a control. (**b**) Lower image: As in (**a**), but in the presence of DMSO (ctrl.) or the NADPH oxidase inhibitor diphenyl iodium salt (DPI, 500 nM). After 28 days, supernatant was transferred to a new 6-well plate and cells were allowed to reattach for 24 h, followed by staining with 2% crystal violet solution. (**c**) N-RAS^61K^ cells were kept for 4 weeks in the absence (ctrl) or presence of doxycycline (Dox) under normoxic (20% O_2_) or hypoxic conditions (1% O_2_), respectively. One sample in each set was treated with doxycycline until senescence was visible, then doxycycline was withdrawn for the remaining time (Dox off). Four weeks after the start of the experiment, supernatant was transferred to a new plate and was stained with 2% crystal violet solution. The experiment was performed three times independently, and the image shows a representative staining. (**d** and **e**) Real-time PCR analysis of *Tyrp1*, *Dct* and *Mlana* (**d**) and *Pdpn* (**e**) from N-RAS^61K^ cells as described in (**c**). Data are derived from two independent experiments, each performed in triplicate. (**f**) RT-PCR analysis of *Nefl* in response to N-RAS^61K^ induction as described in (**c**) (40 cycles). *Hprt* served as a control

Next, we analyzed the pathways necessary for mediating the generation of N-RAS^61K^-AR cells by using inhibitors, which were added to the cells at the start of the experiment. We first blocked the MAPK and PI3K pathways, which are both direct effectors downstream of N-RAS^61K^, using the small molecule inhibitors PD184352 or LY294002, respectively. MEK inhibitor PD184352, but not PI3K inhibitor LY294002 prevented the formation of multinucleated cells (Supplementary Figure S8A). In both cases, the cells were incapable of producing anoikis-resistant progeny (Figure 7a). Similar results were obtained with a different PI3K inhibitor (GDC-0941, Supplementary Figure S8C).

ROS have an important role in melanoma development and pro-tumorigenic signaling.^29, 30, 31^ RAS activation leads to the induction of NADPH oxidase isoforms (Figure 7b, upper image and Weyemi *et al.*^32^). This is accompanied by the generation of ROS.^32, 33^ Accordingly, activation of oncogenic N-RAS in melan-a cells causes DNA damage and p53 activation, as described above. To elucidate the role of DNA damage and senescence induction in N-RAS^61K^-AR development, we applied the antioxidant glutathione ethyl ester or inhibited NADPH oxidase (diphenyle iodonium, DPI), ATM (caffeine) or p53 (pifithrine). DPI, caffeine and pifithrine, but not the glutathione component, were able to prevent the generation of multinucleated (Supplementary Figures S8A and B) and anoikis-resistant cells (Figures 7a and b, lower image), consistent with the involvement of DNA damage and senescence induction in this process. To test whether the inhibitors that prevent anoikis resistance are only involved in preventing senescence or also have a role in allowing later stages of anoikis resistance, we treated N-RAS^61K^ cells with PD184352, LY294002, DPI, pifithrine or caffeine only after senescence was induced (Supplementary Figure S8D). Again, anoikis resistance was prevented, suggesting that the escape from senescence is also be affected by MAPK-PI3K- and DNA damage signaling.

In their tissue of origin, melanocytes encounter hypoxic conditions. We therefore performed a comparative analysis of N-RAS^61K^-induced anoikis resistance and gene regulation under normoxic (20% O_2_) and hypoxic (1% O_2_) conditions. Importantly, N-RAS^61K^ was also able to trigger OIS in hypoxia (Supplementary Figure S9). Similarly, differentiation genes were downregulated (Figure 7d) and the stemness marker *Pdpn* as well as the neuronal marker *Nefl* were strongly upregulated under hypoxic conditions. The cells were also able to undergo anoikis resistance, albeit at lower efficiency compared with N-RAS^61K^ cells kept under normoxic conditions (Figure 7c). To check whether continuous N-RAS^61K^ signaling is only required for the entry into senescence or whether it also affects senescence escape, we additionally investigated the effect of doxycycline withdrawal (thereby switching off N-RAS^61K^). Under normoxic and hypoxic conditions, continuous N-RAS^61K^ signaling was required even after senescence entry to allow all the features of anoikis resistance (Figures 7c–f).

## Discussion

Despite the fact that premalignant tissue often displays features of senescence, senescence is generally considered as a tumor-suppressive mechanism, which either stalls the tumorigenesis of premalignant cells (in case of OIS) or blocks the progression of established tumors (in case of therapy-induced senescence (TIS) of tumor cells). It was recently shown that TIS is induced by a combination of IFN-*γ* and TNF, which leads to impressive control of tumor growth.^34^ However, the idea that senescence exclusively operates as tumor-suppressive mechanism was previously challenged by the observation that, in their niche, senescent cells secrete factors that provide autocrine inhibitory signals, but also stimulate neighboring premalignant cells or cause low levels of tumor-promoting inflammation.^35, 36^ Furthermore, senescent cells harbor epigenetic markers, which are also found in cancer.^37^ Here we provide experimental evidence that OIS can be overcome on the cellular level and describe the generation of highly tumorigenic melanoma cells, characterized by dedifferentiation and stem-like features, from senescent, multinuclear pigment cells.

The accumulation of bi- and multinuclear cells is a common feature of senescent cells *in vitro*, and DNA damage, caused by oncogenic or replicative stress, often mediates senescence.^38^ We observed that the development of N-RAS^61K^-AR cells was prevented by inhibiting senescence mediators such as NADPH oxidases and p53. Importantly, cells that were specifically isolated according to their high p53 expression gave rise to N-RAS^61K^-AR cells, thus indicating that senescence induction is a pre-requisite for the observed anoikis resistance.

The PI3K and MAPK pathways are both required for the development of anoikis-resistance. MEK inhibition of N-RAS^61K^ cells prevented the development of multinucleated cells and consequently AR development. By contrast, PI3K inhibition allowed multinucleation, but prevented anoikis resistance, implicating a role for this pathway at later stages of AR development and highlighting a co-operativity between the MAPK and PI3K pathways in generating tumor-initiating cells from senescent cells. In line with these observations, it was recently shown that high PI3K activity, enabled by PTEN depletion, helps to overcome BRAF^V600E^-induced senescence.^39^

Although we have observed this phenomenon in our experimental setting *in vitro* and clinical confirmation is still missing, the relevance of our findings for animal models and human melanoma is supported by several observations from other groups. First, mice with lung-specific expression of oncogenic RAS first develop senescent pre-malignant lung adenomas, which only later develop into adenocarcinomas.^6^ As all affected lung cells express oncogenic RAS, and this induces senescence, the tumorigenic process is likely initiated after the induction of senescence. Second, staining of the senescence mediator and DNA damage marker p53 is retained in human melanoma with a history of high cumulative sun exposure, and is even associated with higher Breslow thickness,^40^ suggesting residual pro-senescent signaling in melanoma and its compatibility with tumor maintenance. Finally, polyploid cells, which we identified as a source for the malignant offspring, are not only found in melanoma, but also in melanocytic nevi, as shown by us and others,^41, 42^ and a significant proportion of melanomas arise from pre-existing nevi.^43, 44^ In lentigo maligna (LM), also called melanoma *in situ*, multinucleated ‘star burst cells' are present in up to 85% of patient samples.^15^ LM mainly occurs in chronically sun-damaged skin and is associated with UV light exposure as well as p53 positivity,^40, 45^ suggesting a causal relationship between UV-induced DNA damage, which is a strong senescence trigger, and the appearance of starburst cells.

In general, multinucleated or polyploid cells are at increased risk of becoming cancerous through a combination of genomic instability that facilitates the appearance of further mutations^46^ and aberrant gene expression owing to the presence of multiple copies of many genes. Interestingly, irradiated cells of different origin displayed a behavior comparable to our observations, which was termed ‘neosis'.^47^ Here, the neotic cells also adapted a transformed phenotype. The ‘budding' or ‘neotic' behavior strongly resembles the process of ‘depolyploidisation' by asymmetric division, which was recently described for tetra- or polyploid tumor cells. Such cancer cells can undergo a ploidy cycle where aneuploid cells give rise to para-diploid cells, thereby re-aligning to normal cell-cycle regulation and reducing the risk of lethal accumulation of DNA damage.^48, 49^ A shared phenomenon is the upregulation of meiotic genes such as *Spo11,* which is associated with depolyploidisation. This upregulation might reveal a functional parallel between asexual ploidy reduction, for example, occurring in cancer, and sexual ploidy reduction, occurring during meiosis.^50^ Importantly, depolyploidization was also described to go along with increased levels of *OCT4*, *NANOG* and *SOX2*.^47^ Stem cell-like features have also been noted in the progeny of polyploid giant cancer cells,^51^ suggesting that multinucleated senescent cells and polyploid cancer cells share crucial pro-tumorigenic features.

Our data illustrate a mode of overcoming senescence that takes place in response to naturally occurring oncogenes such as the melanoma oncogene N-RAS^61K^. This process is accompanied by the loss of melanocyte markers and by the increase of neuronal- and neural crest-like expression patterns. Importantly, the cells display features of highly dedifferentiated cells, such as a high nuclear-cytoplasmic ratio and increased expression of the transcription factor NANOG, which might explain their aggressive growth and early metastasis *in vivo*. Similar to our observations in melanocytes, transformed pigmented melanoma cells also show the tendency to express enhanced levels of stem cell markers or markers of melanoma initiating cells when they are forced into dedifferentiation,^25, 52^ thus suggesting a common epigenetic and pro-tumorigenic program that can not only be triggered in transformed melanoma cells, but also in senescent melanocytes.

## Materials and Methods

### Cell culture

N-RAS^61K^ and Hm^hi^-cells as well as melan-a cells were presviously described and cultivated as reported earlier.^11, 53^ In addition to these described pTRE2-hyg-N-RAS^61K^ cells, which were derived from one cell clone, another inducible N-RAS^61K^ transgenic cell line was generated. We used the vector pSB-ET-iE (M. Gessler, Dept. of Developmental Biochemistry, University of Wurzburg), which allows integration of N-RAS^61K^ by *sleeping beauty*-mediated transposition. Here, the responsive T6 promoter drives expression of N-RAS^61K^ and EGFP, the latter being separated from the *NRAS* gene by an IRES site. After transposition, these cells were selected with 1 *μ*g/ml puromycin for 2 weeks, and the oligoclonal cell population was used for further experiments. N-RAS^61K^ was cloned into the vector using *Nhe*I/*Afe*I restriction enzyme sites. For long-term treatment, cells were cultured in starving medium (Dulbecco's Modified Eagle's Medium with 10% dialyzed, fetal calf serum (Gibco/Invitrogen, Karlsruhe, Germany) for 3 days before the assay. Induction of the oncogene was generally done using 1 *μ*g/ml doxycycline for the indicated timespans, if not indicated otherwise. In general, medium containing doxycycline was changed 2–3 times per week.

The vectors pBabe-puro-H2-eGFP and pBabe-MN [EF1a-red membrane and green nucleus]-2APuro were delivered into the cells by retroviral infection according to standard protocols. pBabe-puro-H2-eGFP contains a fusion protein of histone 2B and enhanced GFP, thus leading to green fluorescence of the nucleus. pBabe-MN [EF1a-red membrane and green nucleus]-2APuro vector expresses a fusion product of the membrane targeting sequence MGCIKSKRKDNLNDDE with mCherry, followed by a T2A site and a fusion protein of histone 2B with eGFP.

Normal human embryonic melanocytes (NHEM) cells were transfected with pCDH-CMV-NRAS^61K^-EF1-copGFP and were cultivated in melanocyte growth medium (Promocell, Heidelberg, Germany). pGreenFire-p53 (Biocat, Heidelberg, Germany) was delivered into N-RAS^61K^ cells by lipofection according to standard protocols. Where indicated, cells were treated with LY294001, GDC-0941, PD184352 (Selleckchem, Houston, TX, USA), glutathione reduced ethyl ester, pifithrine, caffeine or diphenyliodonium chloride (Sigma-Aldrich, Taufkirchen, Germany).

### RNA isolation and real-time PCR

RNA isolation was performed with TrIR solution (ABGene, Hamburg, Germany) from at least two independent biological replicates. Whole RNA was reversely transcribed using the RevertAidTM First Strand cDNA Synthesis Kit (Fermentas, Leon-Rot, Germany). Fluorescence-based quantitative real-time PCR was performed using the iCycler (Bio-Rad, Munich, Germany), and for each gene, three independent real-time PCRs were performed with cDNA from each biological replicate. The sequence of oligonucleotides used can be found in the Supplementary information (Supplementary Table 3). Gene expression was normalized to hypoxanthine-guanine phosphoribosyltransferase. Relative expression levels were calculated applying REST software.

### Protein blot

Cells were lysed in HEPES-based lysis buffer (20 mM HEPES (pH 7.8), 500 mM NaCl, 5 mM MgCl2, 5 mM KCl, 0.1% deoxycholate, 0.5% Nonidet-P40, 10 mg/ml aprotinin, 10 mg/ml leupeptin, 200 mM Na_3_VO_4_, 1 mM phenylmethanesulphonylfluoride and 100 mM NaF). In all, 30–50 *μ*g of protein lysate was separated by SDS-PAGE and was transferred onto nitrocellulose membranes. Anti-β-actin (C4), NRAS (F155) and p21 (N20) antibodies were purchased from Santa Cruz Biotechnology (Heidelberg, Germany). P19-ARF antibody (#ab80) was from Abcam (Cambridge, UK). Antibodies directed against P-ERK1/2 (Thr202/Tyr204, #9101), P-AKT (Ser473, #4060), P-p53 (Ser18, #9284) and *γ*-H2AX (#2577) were purchased from Cell Signaling Technologies (Danvers, MA, USA). Anti-vinculin antibody (#V-9131) was acquired from Sigma-Aldrich (Heidelberg, Germany). P53 antibody was generated from hybridoma supernatant and was supplied by T. Stiewe (Institute of Molecular Biology and Tumor Research, University of Marburg, Germany). Generally, the presented protein blots are representative for 2–3 independent experiments.

### SA-*β*-Gal staining

Cells were washed with phosphate-buffered saline (PBS; pH 7.2) and fixed with 3.7% formaldehyde in PBS for 5 min at room temperature. After washing, they were stained in the dark for 12 h at 37 **°**C (using 1 mg/ml X-Gal, 40 mM citric acid/sodium phosphate buffer (pH 6.0), 5 mM potassium ferricyanide, 5 mM potassium ferrocyanide, 150 mM NaCl and 2 mM MgCl_2_). Cells were then washed with PBS and were examined by light microscopy.

### Soft agar growth

One milliliter of 1.2% agar was mixed with 1 ml 2 × DMEM supplemented with 10% FCS and plated onto 6-well plates. Upon polymerization, the solid agar was overlain with an equal amount of soft agar mix (0.6% agar) containing 4 × 10^4^ cells per well. The polymerized soft agar was overlain with 100 *μ*l of D10 medium every second day.

### *In vivo* growth

Nude mice (NMRI, Harlan strain) were subcutaneously injected with 2.5 × 10^6^ melan-a, N-RAS^61R^ or N-RAS^61R^-AR cells per flank. Where indicated, 2 mg/ml doxycycline (Sigma-Aldrich) dissolved in 10% saccharose solution was supplied *ad libitum* by administration to the drinking water. After 4 weeks, N-RAS^61R^-AR mice were killed due to considerable tumor load. In all other mice, growth of injected cells was monitored for 10 weeks after injection before killing the mice. All experiments were in accordance with the institution's guidelines.

### Immunohistochemistry

Ki67 and phospho-Histone H3-S^10^ staining was perfomed using SP6 (Neomarkers, Fremont, CA, USA) and 06-570 (Upstate/Milipore, Billerica, MA, USA) as previously described.^54^ Alexa-Fluor conjugated anti-rabbit secondary antibodies (Invitrogen/Life Technologies, Darmstadt, Germany) were used for detection and tissues were counterstained with Hoechst 33342 (Invitrogen).

Paraffine-embedded material of human nevi was subjected to routine histopathological methods. For determination of multinucleated cells, slides were stained with hematoxyline-eosine and in addition with PAS reaction (period acid Schiff reaction), as recommended by the manufacturer (Sigma-Aldrich).

### Microarray

NRAS^61K^ cells were treated for 6, 14 or 28 days with 1 *μ*g/ml doxycycline (Sigma-Aldrich) and NRAS^61K^-AR cells underwent the same treatment for 10 days before being harvested. RNA was isolated using the miRNeasy Kit (Qiagen, Hilden, Germany), and analyzed using the Affymetrix Gene Chip Mouse Genome 430 2.0. All data were analyzed using different R packages from the Bioconductor project (www.bioconductor.org). Obtained data are deposited at http://www.ncbi.nlm.nih.gov/geo/query/acc.cgi?acc=GSE27010.

### Statistic analysis

Generally, all graphs depict the mean values of at least three independent experiments, standard deviations are indicated. Student's *t*-test (two-tailed, paired) revealed statistical significance highlighted by asterisks (*P<0.05; **P<0.01, ***P<0.001).

## Abbreviations

AKT: protein kinase B
AREG: amphiregulin
BRAF: v-Raf murine sarcoma viral oncogene homolog B1
BTC: betacellulin
CYP26B1: cytochrome P450, family 26, subfamily B, polypeptide 1
DCT: dopachrome tautomerase
(E)GFP: (enhanced) green fluorescent protein
EGFR: epidermal growth factor receptor
ERK1/2: extracellular regulated kinase 1/2
FABP4: fatty acid binding protein 4
FLT1: fms-related tyrosine kinase 1 (or vascular endothelial growth factor receptor 1)
*γ*-H2 AX: histone H2AX, phosphorylated at serine 139
HBEGF: heparin binding epidermal growth factor
IFN-*γ*: interferon-*γ*
IRES: internal ribosomal entry site
MAPK: mitogen-activated protein kinase
MITF: microphthalmia-associated transcription factor
MLANA: protein MLANA, (or melanoma antigen recognized by T-cells 1)
NHEM: normal human epidermal melanocytes
N-RAS: neuroblastoma RAS viral oncogene homolog
N-RAS61K-AR: anoikis-resistant cells derived from N-RAS^61K^ transgenic melanocytes
Ki67: intracellular proliferation marker
KITL: KIT ligand (or stem cell factor)
MEK: mitogen-activated protein kinase kinase kinase (or MEK kinase)
NANOG: homeobox transcription factor nanog
NEFL: neurofilament, light polypeptide
OCT4: octamer-binding protein 4
OIS: oncogene-induced senescence
P16: cyclin-dependent kinase inhibitor 2A
P19-ARF: alternate reading frame protein product of the CDKN2A locus
P21: cyclin-dependent kinase inhibitor 1
P53: tumor protein p53
PDPN: podoplanin
PDGFRA: platelet derived growth factor receptor alpha
PI3K: phosphoinositide-3 kinase
PKC: protein kinase C
PTEN: phosphatase and tensin homolog
PTGS2: prostaglandin-endoperoxide synthase 2
RAS: rat sarcoma proto-oncogene
ROS: reactive oxygen species
RRM2: ribonucleotide reductase, subunit M2
SA-*β*-Gal: senescence-associated *β*-galactosidase
SOX2: SRY (sex determining region Y)-box 2 transcription factor
SOX10: SRY (sex determining region Y)-box 10 transcription factor
SPO11: SPO11 meiotic protein covalently bound to DSB (or cancer testis antigen 35)
TIS: therapy-induced senescence
TNF: tumor necrosis factor
TPA: tetradecanoyl-12,13-phorbolacetate
TYRP1: tyrosinase-related protein 1
UV: ultraviolet
VEGFA: vascular endothelial growth factor A
XMRK: *Xiphophorus* melanoma receptor kinase
