# Supplementary material for: In vitro evidence for senescent multinucleated melanocytes as a source for tumor-initiating cells
Source: Cell Death Dis. 2015 Apr 2;6(4):e1711–. doi: 10.1038/cddis.2015.71 (PMC4650540; doi:10.1038/cddis.2015.71)
Supplement: Supplementary Material [file cddis201571x1.pdf]

Supplementary material

Supplementary Figure legends

Supplementary Figure 1: Senescence-associated proliferation stop in response to oncogenic N-RAS expression

Proliferation of NHEM cells without (ctrl.) and with N-RAS^{61K} expression. Cells were seeded at equal density, and the number of cells after 3 and 14 days was determined by manual cell counting. Asterisks indicate statistical significance. *: $p<0.05$. Data are derived from three independent experiments.

Supplementary Figure 2: Senescence features in N-RAS^{61K} cells

A: Total NRAS expression after 4 days of doxycycline induction. Vinculin served as loading control. **B:** Expression of the NRAS downstream components P-ERK1/2 (Thr202/Tyr204) and P-AKT (Ser473) and as well as senescence markers p21, p19^{ARF}, γ-H2AX (Ser139), P-p53(Ser18), and p53 after induction of oncogene induced senescence in N-RAS^{61K} cells. Vinculin served as loading control. **C:** Small cells appear in close proximity to multinucleated senescent cells. Cells were seeded at very low density and were stimulated with doxycycline for two weeks. Scale bar=75 µm.

Supplementary Figure 3: N-RAS^{61K}–AR cells show aberrant growth behavior even in presence of reduced serum.

A: Comparison of cellular appearance of melan-a control, N-RAS^{61K} and N-RAS^{61K}–AR cells after 14 days (14d) of treatment in absence or presence of doxycycline (Dox) treatment (1 µg/ml) (phase contrast). Scale bars, 100 µm. **B:** Proliferation of melan-a (ctrl.), N-RAS^{61K} and N-RAS^{61K}-AR cells in DMEM containing Dox and 2.5% or 10% FCS (dialyzed), as indicated. Asterisks indicate statistical significance of N-RAS^{61K}-AR compared to N-RAS^{61K} cells. *: $p<0.05$; **: $p<0.01$, ***: $p<0.001$; n=3.

Supplementary Figure 4: Melan-a and Melan-a-N-RAS^{61K} cells are not tumorigenic *in vivo*.

A: Macroscopic appearance of subcutaneous tissue 10 weeks after injection of melan-a control cells into nude mice. **B:** subcutaneous accumulation of injected melan-a cells forming a nevus-like structure (scale bar, 50 µm). **C:** Hoechst 33342 (Hoechst) and Ki67 staining of a tissue section through the skin of melan-a injected mice. Scale bars, 500 µm. **D:** Hoechst and phospho-histone H3 (P-H3) staining of a tissue section through the skin of melan-a injected mice. Scale bars, 500 µm. Melan-a cells, identifiable by their brown appearance in the unstained state, are negative for Ki67 and P-H3.

Supplementary Figure 5: Proliferation genes are induced by N-RAS^{61K} expression.

A: Heatplot displaying expression levels of proliferation genes in N-RAS^{61K} cells stimulated with doxycycline for 6, 14, and 28 days and in N-RAS^{61K}–AR cells. The values are color coded using a green–red scale, where green is low expression and red is high expression. **B:** Real-time PCR analysis of *Flt1* and *Btc* expression and RT-PCR analysis of *Ptgs2* expression (40 cycles). *Hprt* served as control. **C:** Western blot showing the levels of activated ERK1/2 (indicated by phosphorylation at Thr202/Tyr204) in N-RAS^{61K} and N-RAS^{61K}-AR cells after cultivation in TPA-free medium with 10% FCS (D10), starving medium ("starv.": TPA-free, 10% dialyzed FCS) or starving medium containing doxycycline for indicated time points (4d, 6d, 14d, 28d). β-actin served as loading control. **D:** Heatplot displaying the RNA expression of meiosis genes in N-RAS^{61K} cells stimulated with doxycycline for 6, 14, and 28 days and in N-RAS^{61K}–AR cells. The values are color coded using a green–red scale, where green is low expression and red is high expression. **E:** Confirmation of differential gene expression by real-time PCR using primers directed against *Cyp26b1* and *Spo11*.

Supplementary Figure 6: Dose-dependent induction of N-RAS^{61K}

A: Phase contrast (left) and GFP (right) images of pSB-N-RAS^{61K} cells which were kept for one week in presence of the indicated doxycycline concentration. B: RT-PCR analysis of *Nras* in response to N-RAS^{61K} induction as described in **A** (30 cycles). *Hprt* served as control. Please note that the *Nras* oligonucleotides also recognize endogenous *Nras*. Bars indicate 100 µm.

Supplementary Figure 7: Anoikis resistance occurs independently of *Nra*s expression level

A: Phase contrast images (PH) and SA-β-Gal stainings of pSB-N-RAS^{61K} cells which were kept for 11 days in presence of the indicated doxycycline concentration. Bars indicate 100

μm. **B:** N-RAS^{61K} cells were cultivated for 4 weeks in presence of indicated doxycycline concentrations. Afterwards, supernatant was transferred to a new 6-well plate, and crystal violet staining was performed. **C, D:** Real-time PCR analysis of *Tyrp1*, *Dct*, and *Mlana* (**C**) and *Pdpn* (**E**) from N-RAS^{61K} cells cultivated for four weeks in presence of the indicated concentrations of doxycycline. Data are derived from two independent experiments, each performed in triplicate **E:** RT-PCR analysis of *Nefl* in response to N-RAS^{61K} induction as described in **C** (40 cycles). *Hprt* served as control.

Supplementary Figure 8: Inhibition of PI3K, MEK, p53, ATM and NADPH oxidases prevents anoikis resistance development.

A: N-RAS^{61K} cells were cultivated for 28 days in presence of doxycycline (Dox) and DMSO (ctrl.), the PI3K inhibitor LY294002 (LY, 10 μM), the MEK inhibitor PD184352 (PD, 2 μM), the antioxidant glutathione reduced ethyl ester (GRE, 1 mM), the p53 inhibitor pifithrine (Pifi, 10 μM), or the ATM inhibitor caffeine (Caf, 1 mM). **B:** N-RAS^{61K} cells were cultivated for 28 days in presence of doxycycline and DMSO (ctrl.) or the NADPH oxidase inhibitor diphenyl iodium salt (DPI, 500 nM). Pictures were taken after 8 and 28 days of treatment. Scale bars, 100μm. **C:** As in **A** and **B**, but in presence of DMSO (ctrl.) the MEK inhibitor PD184352 (PD, 2 μM), or the PI3K inhibitors LY294002 (LY, 10 μM) or GDC-0941 (3 μM), respectively. After 28 days, supernatant was transferred to a new 6-well plate and cells were allowed to reattach for 24 h, followed by staining with 2% crystal violet solution. **D:** N-RAS^{61K} cells were cultivated until senescence in presence of doxycycline (Dox). When senescence was reached, cells were additionally treated with DMSO or the indicated inhibitors (DMSO (ctrl.), PD184352 (PD, 2 μM), LY294002 (LY, 10 μM), diphenyl iodium salt (DPI, 500 nM), pifithrine (Pifi, 10 μM), caffeine (Caf, 1 mM)). All agents were replaced twice weekly. Four weeks after the start of the experiment, supernatant was transferred to a new plate and was stained with 2% crystal violet solution. Scale bars, 100μm.

Supplementary Figure 9: Senescence occurs under hypoxic conditions.

Cells were kept for two weeks in hypoxic conditions (1% O_2), before SA-β-Gal staining was performed. Controls displayed no sign of senescence, and thus the phase contrast (PH) is shown to visualize cell borders. Scale bars, 100μm.

Supplementary figure 1

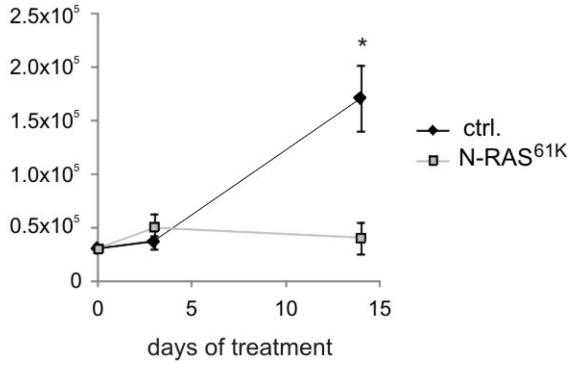

Supplementary figure 2

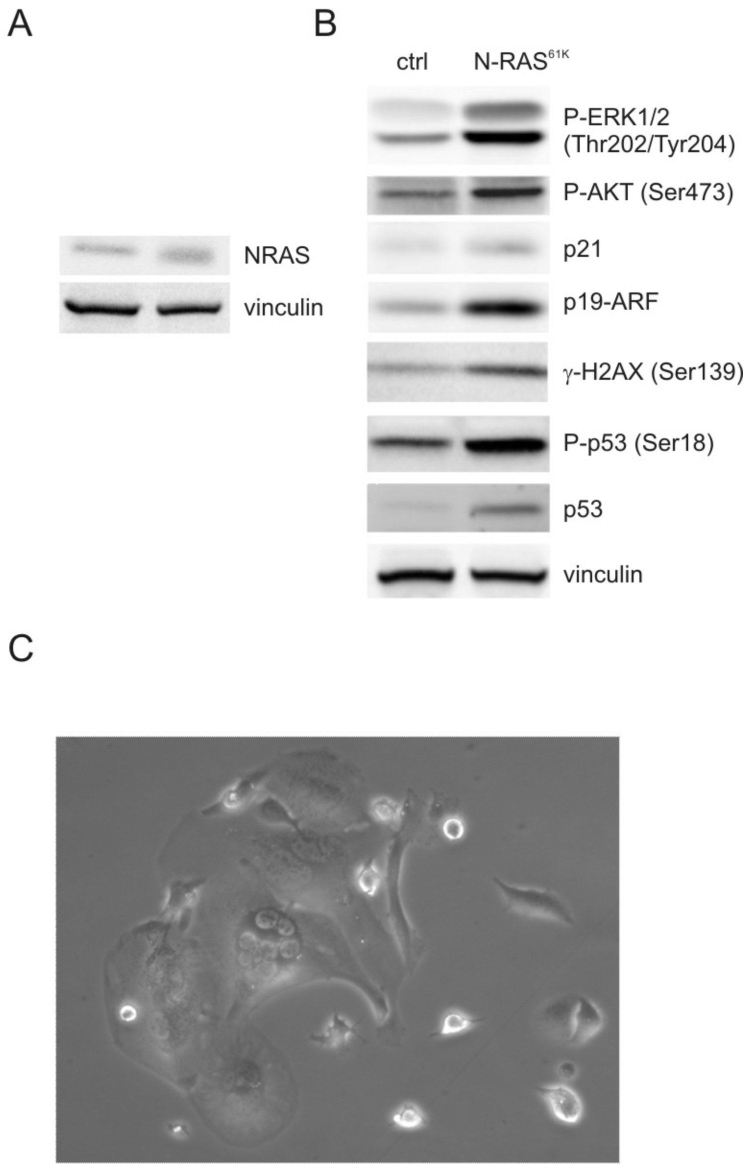

Supplementary figure 3

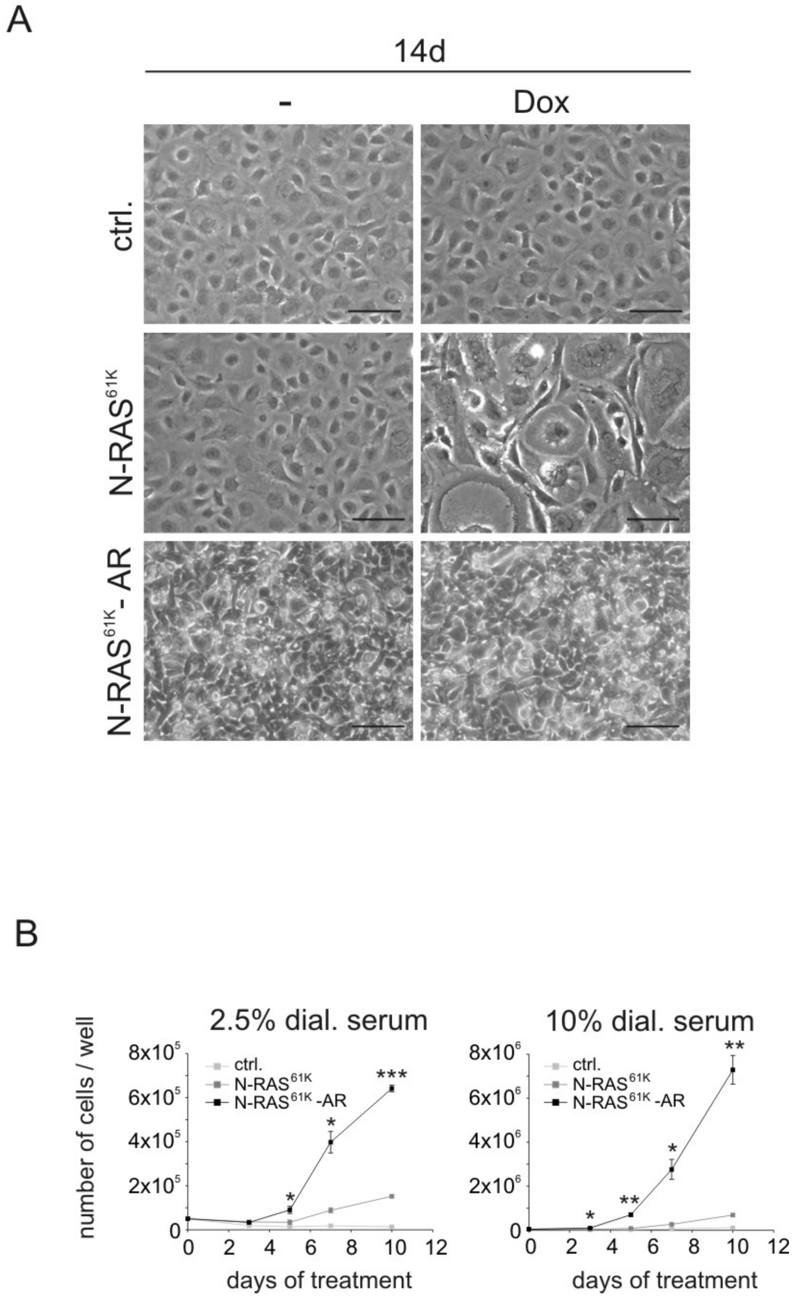

Supplementary figure 4

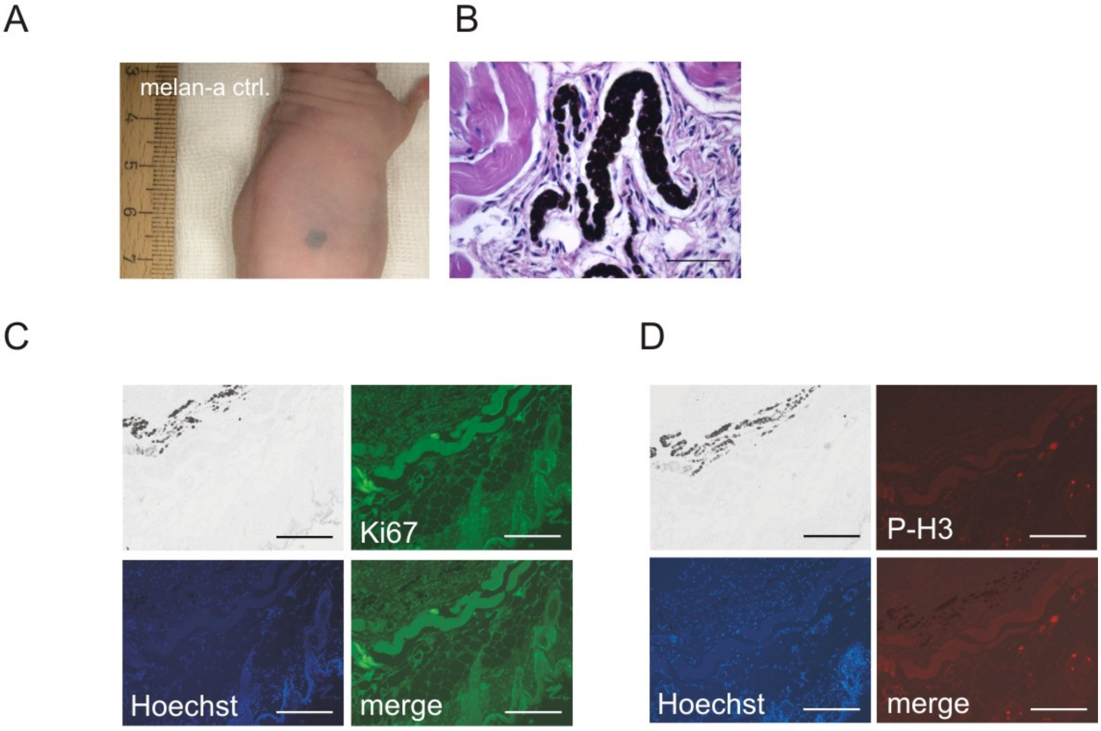

Supplementary figure 5

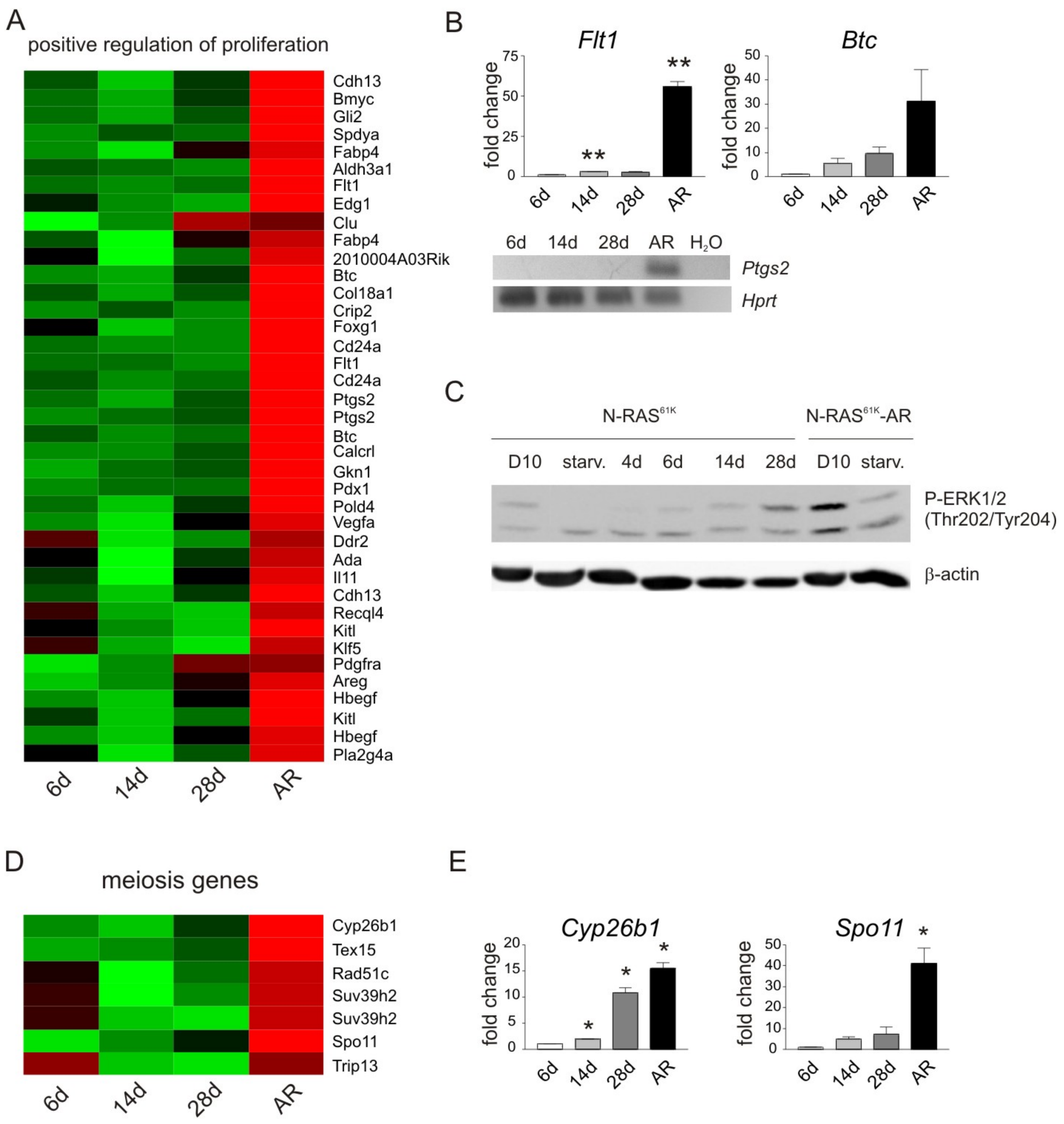

Supplementary figure 6

A

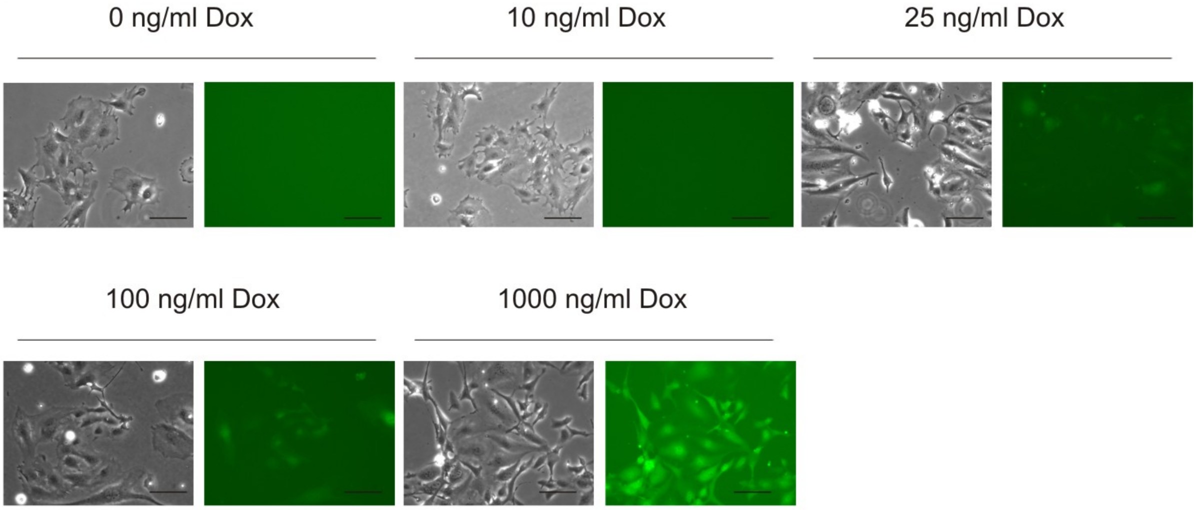

B

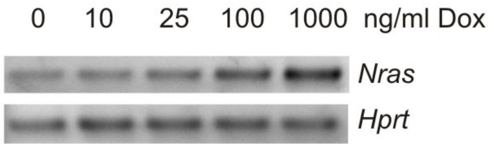

Supplementary figure 7

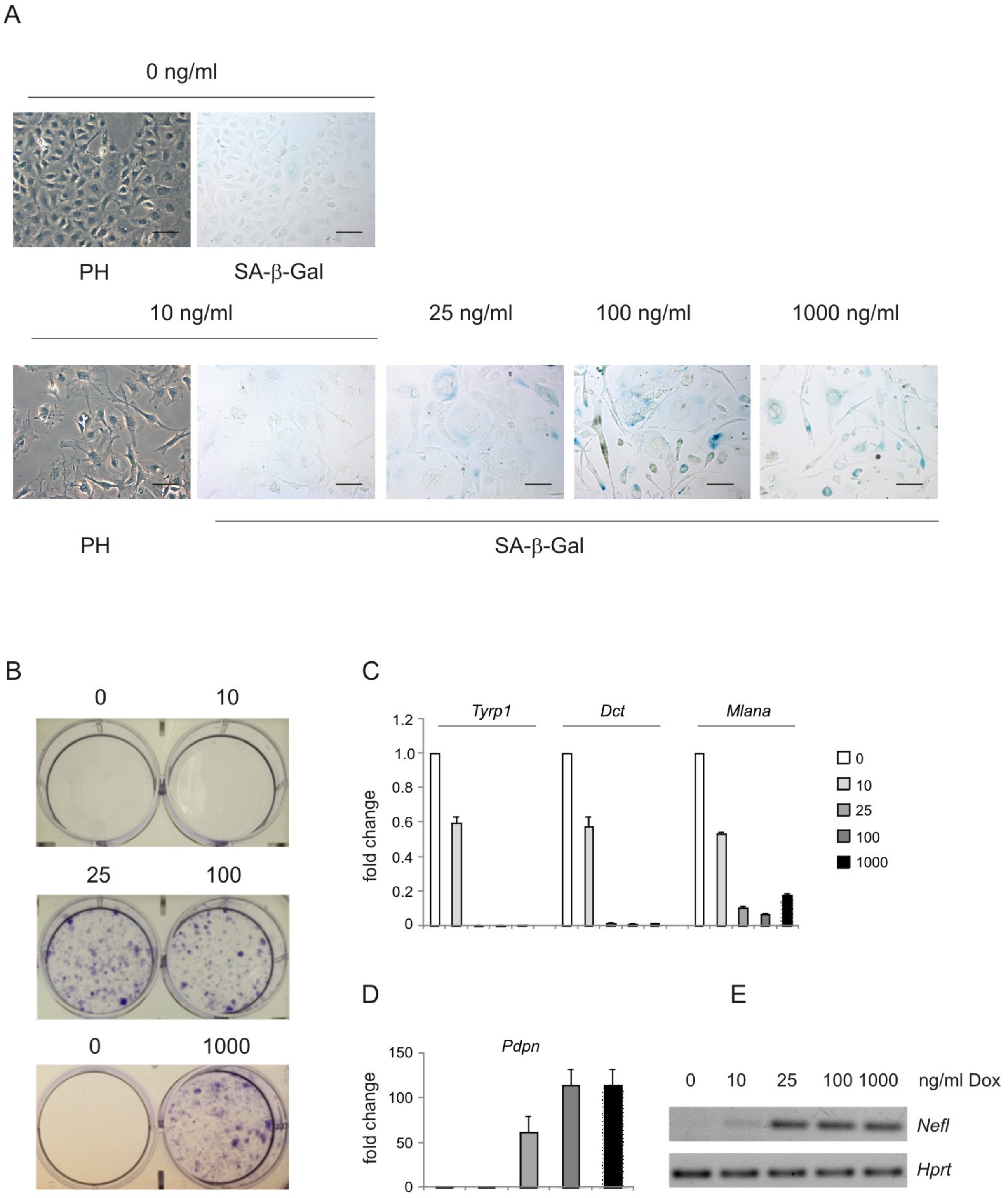

Supplementary figure 8

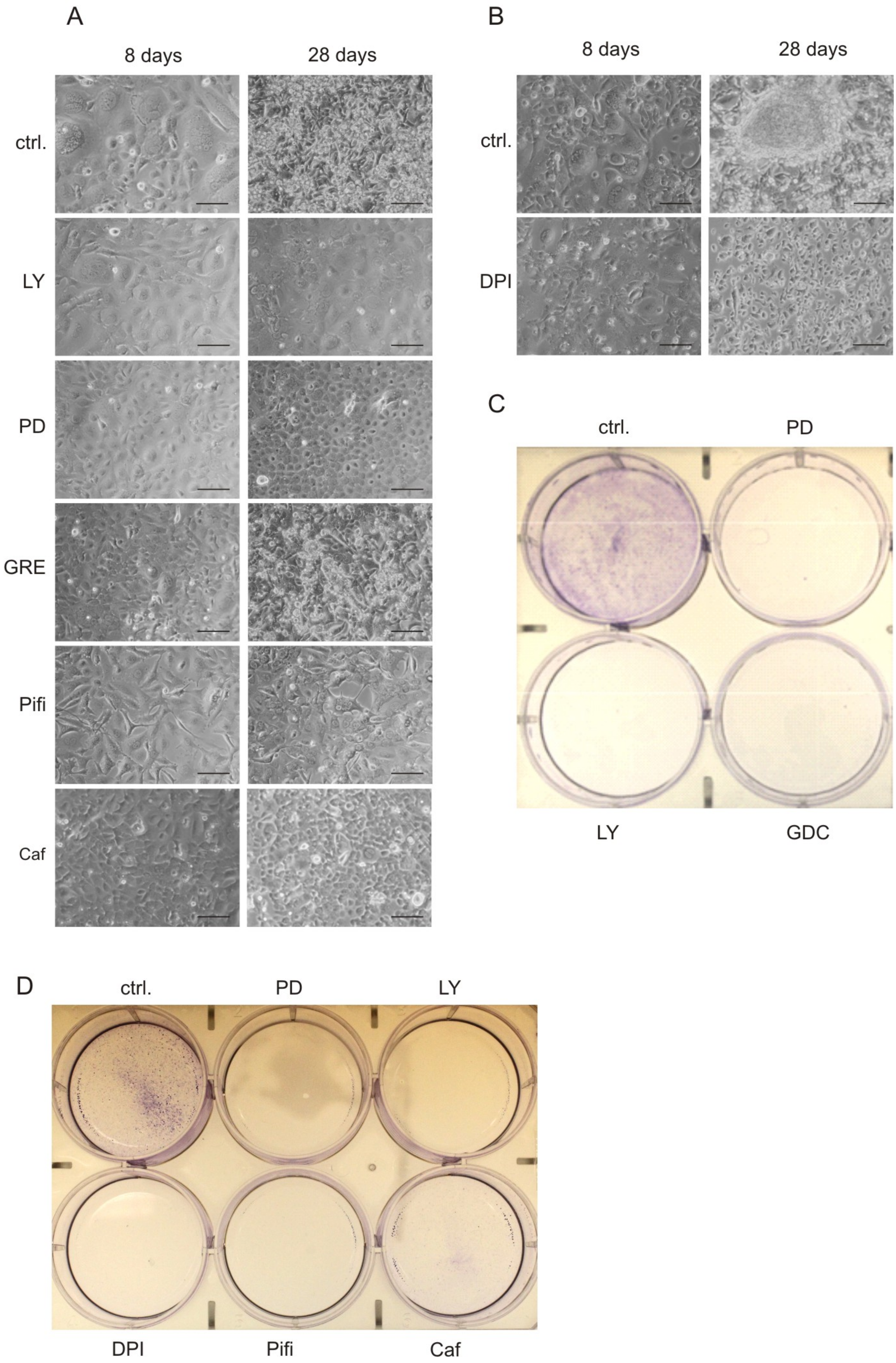

Supplementary figure 9

hypoxia

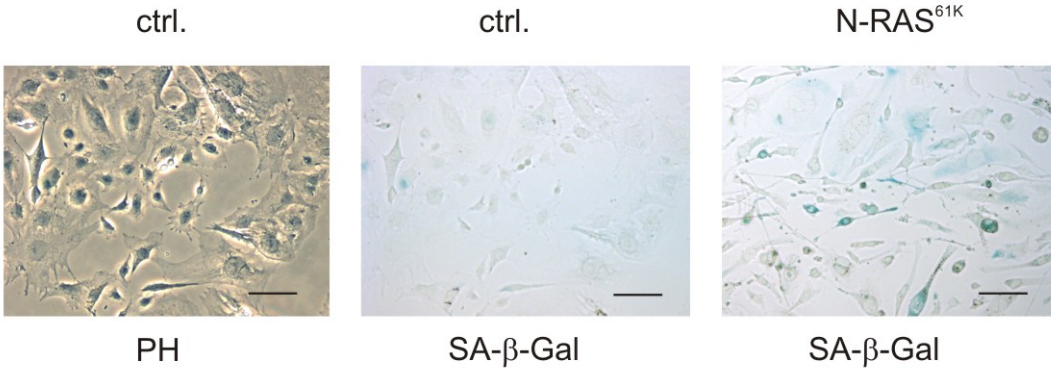

Supplementary Movie 1: Small resistant cells arise from multinucleated cells.

N-RAS^{61K} cells were transiently transfected with pBabe-MN [EF1a-red membrane and green nucleus]-2APuro before being plated onto glass cover-slips. Upon 16 days of doxycycline treatment, cells were monitored for 17 hours at a 100-fold magnification and pictures were taken every 15 minutes. Scale bar, 50µm. 7fps.

Supplementary Movie 2: Cells budding from multinucleated cells are capable of dividing.

N -RAS^{61K} cells were transiently transfected with pBabe-puro-H2-eGFP before being plated onto glass cover-slips. Upon 27 days of doxycycline treatment, cells were monitored for 28 hours at a 100-fold magnification and pictures were taken every 15 minutes. Scale bars, 50µm. **A:** GFP fluorescence of the cells. **B:** As in **A,** but merge of phase contrast and GFP. Time points are indicated. Arrows pinpoint the budding and dividing cell.

Supplementary Movie 3: Increased nuclear/cytoplasmic ratio.

Confocal stacks of N-RAS^{61K} (left) and N-RAS^{61K}–AR cells (right). Arrows, 50 µm.

Supplementary tables:

Supplementary Table 1: Chromosomal aberrations of N-RAS^{61K}-AR cells.

Table displaying gains and losses of chromosomes from four different N-RAS^{61K}-AR cell clones in comparison to their parental untreated N-RAS^{Q61K} cells. The chromosomes were assigned by spectral karyotyping.

Table S1: Chromosome copy alterations in N-RAS^{Q61K} cells

Chromosome	N-RAS^{Q61K}-AR #1	N-RAS^{Q61K}-AR #2	N-RAS^{Q61K}-AR #3	N-RAS^{Q61K}-AR #4
1	-	-	-	-
2	-	-	-	-
3	-	-	-	-
4	+1	+1	-	+1
5	-	-	-	-
6	-	+2	-	-
7	-	-	-1	-
8	-	-	-	-
9	-	-1	-	-
10	-	-	-	-
11	-	-	-	-
12	-	-	+1	+1
13	-	+3	-	-
14	-	-	-	-
15	-	-	-	-
16	-	-1	-1	-
17	-	-	-	-
18	-	-1	-	-
19	-	-	-	-
X	-	-	-	-

Supplementary Table 2: Tumor development in nude mice.

The table summarizes the results of tumor development after injection of indicated cells into the flanks of nude mice.

	melan-a pTREhyg2	melan-a N-RAS^{61K}	melan-a N-RAS^{61K} + Dox	melan-a N-RAS^{61K}- AR
nr of mice (both flanks injected)	5	5	5	5
nr of cells injected per site	$2.5x10^6$	$2.5x10^6$	$2.5x10^6$	$2.5x10^6$
end of experiment (weeks)	10	10	10	4
nr of tumors observed	0 (0%)	0 (0%)	0 (0%)	10 (100%)

Supplementary Table 3: Oligonucleotides used in this manuscript.
The table summarizes the DNA sequences of the oligonucleotides used for real-time and RT-PCR analyses throughout the manuscript.

gene	ENSEMBL reference	forward 5`->3`	reverse 5`->3`
Btc	ENSMUSG00000082361	GCATAGAGAAGGAACCTGAGGACT	GTTTCTGGTGTTCTGGTTGTGTTC
Cyp26b1	ENSMUSG00000063415	TTCTCTCTGCCAGTGGACCT	GGTCATCTCCTTGCCATGTT
Dct	ENSMUSG00000022129	AGCAGACGGAACACTGGACT	GCATCTGTGGAAGGGTTGTT
DCT	ENSG00000080166	GGTTCCTTTCTTCCCTCCAG	AACCAAAGCCACCAGTGTTC
Flt1	ENSMUSG00000029648	CGGCAGACCAATACAATCCT	CCGCTGCCTTATAGATGCTC
Hprt	ENSFM00250000001478	TGTTGTTGGATATGCCCTTG	ACTGGCAACATCAACAGGACT
Mitf	ENSMUSG00000035158	GGAACAGCAACGAGCTAAGG	TGATGATCCGATTCACCAGA
Mlana	ENSMUSG00000024806	ATGTGAGAGCCCTGATCACC	CAGCGTTCTCAGGAGTTTCC
Nanog	ENSMUSG00000012396	AAGTACCTCAGCCTCCAGCA	GTGCTGAGCCCTTCTGAATC
Nefl	ENSMUSG00000022055	CCATGCAGGACACAATCAAC	CGCCTTCCAAGAGTTTTCTG
Nox1	ENSMUSG00000031257	CTGCTCATTTTGCAACCGTA	AGAAGCGAGAGATCCATCCA
Nox2	ENSMUSG00000015340	GCTGGGATCACAGGAATTGT	CTTCCAAACTCTCCGCAGTC
NRAS	ENSG00000213281	GAATATGATCCCACCATAGAG	GCGGCTAGCTTACATCACCACACATGGCAAT
Pdpn	ENSMUSG00000028583	GCCAGTGTTGTTCTGGGTTT	AGAGGTGCCTTGCCAGTAGA
Ptgs2	ENSMUSG00000032487	GCTCTTCCGAGCTGTGCT	GGATTGGAACAGCAAGGATTT
Smarca1	ENSMUSG00000031099	AGCTGGGTACAGTGGAATGG	TCCTTTTCCAGGAGCTCAAA
Sox10	ENSMUSG00000033006	TGGGGATGTGGATTTCTCTC	TGGATCCCATCAAGTCATCA
Spo11	ENSMUSG00000005883	GAGCGACACTTATGCAACCA	CTCAAGTTGCCAGCAATCAA
Tyrp1	ENSMUSG00000005994	TCTGGCCTCCAGTTACCAAC	TCAGTGAGGAGAGGCTGGTT
TYRP1	ENSMUSG00000022129	CCGAAACACAGTGGAAGGTT	TCTGTGAAGGTGTGCAGGAG
